# Preimmunization of donor lymphocytes enhances antitumor immunity of autologous hematopoietic stem cell transplantation

**DOI:** 10.1002/cam4.117

**Published:** 2013-09-10

**Authors:** Koji Suzuki, Kouichirou Aida, Reina Miyakawa, Kenta Narumi, Takeshi Udagawa, Teruhiko Yoshida, Yusei Ohshima, Kazunori Aoki

**Affiliations:** 1 Division of Gene and Immune Medicine, National Cancer Center Research Institute5-1-1 Tsukiji, Chuo-ku, Tokyo, 104-0045, Japan; 2 Department of Pediatrics, Fukui University School of Medicine23-3 Shimoaizuki, Matsuoka, Yoshida-gun, Fukui, 910-1193, Japan; 3 Division of Genetics, National Cancer Center Research Institute5-1-1 Tsukiji, Chuo-ku, Tokyo, 104-0045, Japan

**Keywords:** Donor, gene therapy, hematopoietic stem cell transplantation, interferon-alpha, preimmunization

## Abstract

Lymphopenia-induced homeostatic proliferation (HP) of T cells following autologous hematopoietic stem cell transplantation (HSCT) skews the T-cell repertoire by engaging tumor-associated antigens (TAAs), leading to an induction of antitumor immunity. Here, as the tumor-reactive lymphocytes preferentially proliferate during the condition of HP, we examined whether the priming of a donor lymphocytes to TAAs could enhance HP-induced antitumor immunity in autologous HSCT recipients. First, to examine whether the tumor-bearing condition of donor influences the antitumor effect of HSCT, the lymphocytes isolated from CT26 tumor-bearing mice were infused into lethally irradiated mice. The growth of tumors was substantially suppressed in the mice that received HSCT from a tumor-bearing donor compared with a naïve donor, suggesting that a fraction of donor lymphocytes from tumor-bearing mice are primed in response to TAAs and remain responsive upon transplantation. We previously reported that type I interferon (IFN) maturates the dendritic cells and promotes the priming of T cells. We then investigated whether the further priming of donor cells by IFN-α can strengthen the antitumor effect of HSCT. The intratumoral IFN-α gene transfer significantly increased the number of IFN-γ-positive lymphocytes in response to CT26 cells but not the syngeneic lymphocytes in donor mice. The infusion of primed donor lymphocytes markedly suppressed the tumor growth in recipient mice, and cured 64% of the treated mice. Autologous HSCT with the infusion of primed donor lymphocytes is a promising strategy to induce an effective antitumor immunity for solid cancers.

## Introduction

It was generally assumed that the autologous nature of a graft precludes any immune-mediated antitumor effect, and autologous hematopoietic stem cell transplantation (HSCT) has been performed after intensive chemotherapy to rescue the bone marrow (BM) cells from myeloablative damage^1^. However, several recent preclinical studies reported that immune reconstitution of autologous HSCT recipients promotes T-cell priming and induces antitumor efficacy^2^–^4^. Lymphopenia is followed by spontaneous expansion of the remaining T cells in the periphery to restore the original T-cell pool size and maintain homeostasis. Lymphopenia-induced homeostatic proliferation (HP) of T cells following autologous HSCT is driven by the recognition of self-antigens, and there is an opportunity to skew the T-cell repertoire during the T-cell recovery by engaging tumor-associated antigens (TAAs), leading to an induction of antitumor immunity^5^,^6^. In addition, myeloablative preconditioning should be useful in making space for the expansion of specific antitumor T cells^7^. Furthermore, we recently found that the frequency of regulatory T cells (Tregs) was clearly decreased within the tumors after HSCT^8^, suggesting that autologous HSCT can create an environment strongly supporting the enhancement of antitumor immunity.

Type I interferon (IFN) has important roles in regulating the innate and adaptive immune system: upregulation of major histocompatibility complex class I gene, promotion of the priming and survival of T cells, enhancement of humoral immunity, increase of the cytotoxic activity of natural killer cells and CD8^+^ T cells, and maturation and activation of dendritic cells (DCs)^9^–^11^. To load the antitumor immunity of HSCT with a tumor-specific immune response induced by IFN, we combined intratumoral IFN gene transfer in the early period after syngeneic HSCT. The combination therapy was able to induce a significant systemic antitumor immunity, resulting in the inhibition of subcutaneous tumor growth and suppression of metastasis formation at distant sites such as lung and liver^12^,^13^; however, it was rare to cure tumor-bearing mice. As the tumor-reactive lymphocytes preferentially proliferate during the condition of HP, due to the synergic effect with encountering their cognate antigens in tumor-bearing host^14^, we examined whether the promotion of donor priming status to TAAs enhances the antitumor immunity in HSCT recipient mice. In this study, we found that the preimmunization of donor lymphocytes by intratumoral IFN gene transfer was highly effective in enhancing the antitumor immunity of HSCT, and induced eradication of unmodified tumors.

## Material and Methods

### Animals and HSCT

Seven-to-nine week-old female BALB/c (H-2^d^, Ly-1.2) mice were purchased from Charles River Japan, Inc. (Kanagawa, Japan) and were housed under sterilized conditions. Animal studies were carried out according to the Guideline for Animal Experiments of the National Cancer Center Research Institute and approved by the Institutional Committee for Ethics in Animal Experimentation. BALB/c mice received a lethal dose (9 Gy) of total body irradiation on the day of transplantation. The irradiated BALB/c mice were injected subcutaneously with 1 × 10^6^ of CT26 cells and then intravenously with 5 × 10^6^ of BM cells and 2 × 10^6^ of splenic T cells from donor BALB/c mice. The transfer of T cells (splenocytes) is crucial to induce an antitumor immunity^4^,^8^. BM cells were isolated from donors by flushing each femur and tibia with an RPMI-1640 medium (Nissui Pharmaceutical Co., Tokyo, Japan) supplemented with 5% heat-inactivated fetal bovine serum (FBS) (ICN Biomedicals, Inc., Irvine, CA), and splenic cells were prepared by macerating the spleens. After lysis of the erythrocytes, the splenic cells were incubated with anti-Thy-1.2 immunomagnetic beads (Miltenyi Biotec, Bergisch Gladbach, Germany) at 4°C for 15 min, followed by selection of T cells by AutoMACS (Miltenyi Biotec). The tumor volume was calculated using the formula: tumor volume = 1/2 × ([the shortest diameter]^2^ × [the longest diameter]). Data are presented as mean ± standard deviation (SD). The tumor volume in each group was measured until the statistical significance was detected more than four times, and after that the survival of mice was just followed.

### Tumor cell lines and recombinant adenovirus vectors

CT26 cells (American Type Culture Collection, Rockville, MD) are weakly immunogenic BALB/c-derived colon cancer cell lines. They were maintained in Roswell Park Memorial Institute 1640 (RPMI) containing 10% FBS, 2 μmol/L l-glutamine, and 0.15% sodium bicarbonate (complete RPMI). The recombinant adenovirus vectors expressing mouse interferon-α (Ad-mIFN) and alkaline phosphatase (Ad-AP) cDNA were prepared as described^15^. The recombinant adenoviruses are based on serotype 5 with deletions of the entire E1 and a part of the E3 regions, and have the CAG promoter, which is a hybrid of the cytomegalovirus immediate early enhancer sequence and the chicken β-actin/rabbit β-globin promoter. A cesium chloride-purified virus was desalted using a sterile Bio-Gel P-6 DG chromatography column (Econopac DG 10; BioRad, Hercules, CA) and diluted for storage in a 13% glycerol/PBS (phosphate buffered saline) solution. All viral preparations were confirmed by polymerase chain reaction assay to be free of E1^+^ adenovirus.

### In vivo tumor inoculation and IFN-α gene transfer

CT26 cells (1 × 10^6^) were injected subcutaneously into the legs of BALB/c mice. When a subcutaneous tumor was established (∼1.0 cm in diameter), it was directly injected once with 5 × 10^7^ PFU of Ad-mIFN or control vector (Ad-AP). Two weeks after the virus injection, tumor volume was measured, and the mice were sacrificed to harvest the spleens and BMs as donors.

### Tetramer staining and fluorescence-activated cell sorting analysis

The CT26-specific H-2L^d^ MuLVgp70 (AH-1) peptide tetramer and fluorescein isothiocyanate (FITC)-anti-CD8 antibody were purchased from BD Biosciences. For cell staining, the manufacturer's protocol was followed. Spleens were harvested from the mice, and after washing, the cells were incubated with tetramer and antibody in a total volume of 100 μL PBS with 5% FBS for 30 min at 4°C and then fixed. Cells were analyzed by fluorescence-activated cell sorting (FACSCalibure; BD Biosciences, San Jose, CA). Irrelevant IgG mAbs were used as a negative control. Ten thousand live events were acquired for analysis.

### ELISpot assays

IFN-γ ELISpot kit (BD Biosciences) were used according to the manufacturer's instructions. Briefly, splenocytes- (1 × 10^5^) and mitomycin C (MMC)-treated tumor cells (1 × 10^4^) were cocultured in 96-well plates precoated with mouse IFN-γ (BD Biosciences) for 20 h at 37°C in complete RPMI medium in triplicate. After washing wells, biotinylated anti-mouse IFN-γ antibody (2 μg/mL) was added and incubated for 2 h at room temperature. A streptavidin–horseradish peroxidase solution was then added and incubated for 1 h at room temperature. After the addition of an aminoethyl carbozole substrate solution, spots were counted under a stereomicroscope.

### Statistical analysis

Comparative analyses of the data were performed by the Student's *t*-test, using SPSS statistical software (SPSS Japan Inc., Tokyo, Japan). *P* < 0.05 was considered as a significant difference.

## Results

### Antitumor effect of syngeneic HSCT

**Figure 1:**
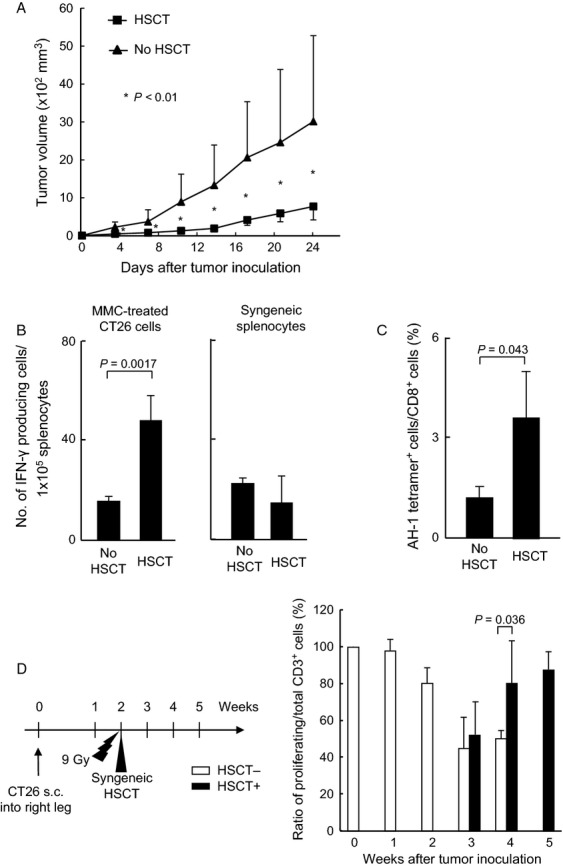
Syngeneic HSCT suppressed the growth of subcutaneous tumors. (A) Growth suppression of subcutaneous tumors in syngeneic HSCT mice. The BALB/c mice received a lethal dose of irradiation, followed by a infusion of BM and splenic T cells derived from BALB/c mice. CT26 cells were inoculated into right legs (number of animals per each group: *n* = 7). Tumor volumes were measured at the indicated days. (B) The increase of IFN-γ-positive cells in response to stimulation of CT26 cells by an ELISpot assay. Two weeks after HSCT, splenocytes were isolated from HSCT mice, and cocultured with CT26 cells or control lymphocytes (*n* = 3). (C) The increase of tumor-specific CD8^+^ T cells in HSCT mice. The splenocytes were isolated 2 weeks after HSCT, and CT26-specific AH-1-tetramer-positive cells were analyzed by flow cytometry (*n* = 4). CD8^+^ lymphocyte regions in FACS plots were gated and developed spots in two dimensions, and the ratio of tetramer^+^ CD8^+^ cells per total CD8^+^ cells was calculated. (D) The increase of proliferating activity of CD3^+^ cells in HSCT mice. HSCT was performed 2 weeks after tumor inoculation, and the proliferation of CD3^+^ T cells was analyzed during 3–5 weeks after tumor inoculation (1–3 weeks after HSCT). The splenocytes were isolated and 5 × 10^6^ of carboxyfluorescein succinimidyl ester (CSFE)-labeled splenocytes were cultured with CD11c^+^ cells in CD3-coated 24-well plates. After 48 h, the proliferating fraction of CD3^+^ cells was evaluated with anti-CD3^+^ antibody (BD Biosciences) by flow cytometry (*n* = 3). The proliferating cells were defined as a single or more round of division. HSCT, hematopoietic stem cell transplantation; BM, bone marrow; IFN, interferon; FACS, fluorescence-activated cell sorting.

To examine whether HSCT could induce antitumor immunity, BALB/c mice were injected subcutaneously with CT26 colon cancer cells shortly after lethal irradiation, and then BM and T cells were infused into the mice to avoid the direct effect of radiation on the tumor cells. Tumor growth was significantly suppressed in the syngeneic HSCT recipients (Fig. 1A) as previously reported^12^,^13^. No overt toxicity was observed for the treated mice, including their blood chemistry. An ELISpot assay showed that HSCT increased the number of IFN-γ^+^ spots in response to CT26 cells but not to syngeneic lymphocytes (Fig. 1B), and the tetramer assay also showed that the frequency of CT26-specific AH-1 responsive CD8^+^ T cells was significantly increased in HSCT recipient mice (Fig. 1C). The results suggested that HSCT was able to induce an antitumor immunity during the process of the HP after HSCT.

It is known that tumor progression results in systemic immune suppression possibly due to the induction of immunosuppressive cells and cytokines^16^–^18^. To examine how syngeneic HSCT affects the systemic immune suppression in tumor-bearing mice, HSCT was performed 2 weeks after tumor inoculation, and the proliferation of CD3^+^ T cells was analyzed during 3–5 weeks after tumor inoculation (1–3 weeks after HSCT). The activity of lymphocyte proliferation decreased in non-HSCT control mice, whereas the proliferation activity increased gradually after 3–5 weeks after tumor inoculation (1–3 weeks after HSCT), and there is a significant difference of lymphocyte proliferation in HSCT mice compared with non-HSCT mice at 4 weeks after tumor inoculation (2 weeks after HSCT) (Fig. 1D). The non-HSCT mice died 5 weeks after tumor inoculation due to the tumor progression. The results suggested that the syngeneic HSCT overcomes the tumor-induced systemic immunosuppression.

### Donor lymphocytes isolated from tumor-bearing mice enhanced antitumor immunity of HSCT

**Figure 2:**
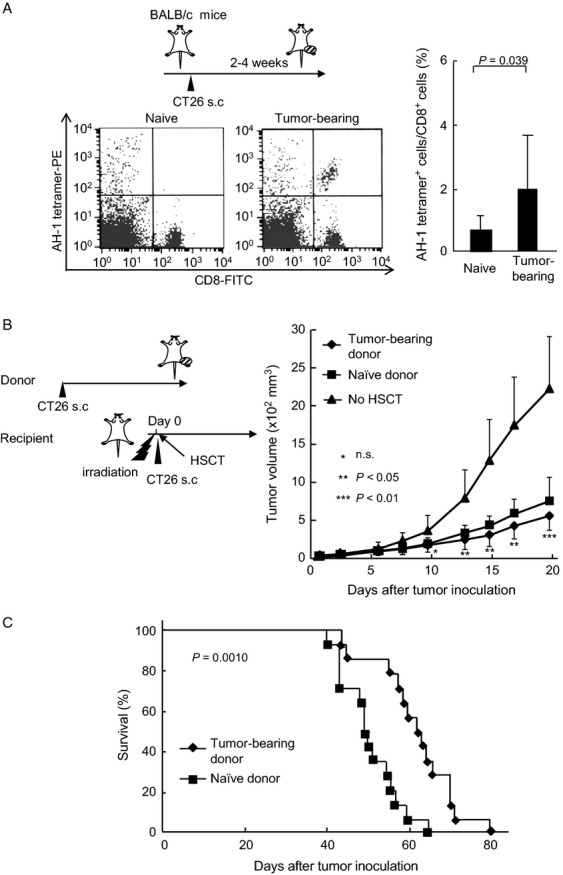
Donor lymphocytes isolated from tumor-bearing mice enhanced antitumor immunity of HSCT. (A) The increase of tumor-specific CD8^+^ T cells in tumor-bearing mice. The splenocytes were isolated 2 weeks after tumor inoculation, and CT26-specific AH-1-tetramer-positive cells were analyzed by flow cytometry (*n* = 4). (B) Growth suppression of subcutaneous tumors by HSCT with lymphocytes isolated from tumor-bearing mice. The lethally irradiated BALB/c mice were infused with BM and splenic T cells derived from naïve (*n* = 11) or tumor-bearing BALB/c mice (*n* = 13). CT26 cells were inoculated into right legs. The statistical significances between tumor volumes in HSCT with naïve donors and those with tumor-bearing donors are presented. (C) HSCT with lymphocytes isolated from tumor-bearing mice extended the survival of recipient mice. The BM and splenic T cells were isolated from naïve (*n* = 11) or tumor-bearing BALB/c mice (*n* = 13). HSCT, hematopoietic stem cell transplantation; BM, bone marrow.

Although the recipient immune system was reconstituted by the naïve donor lymphocytes in Figure 1A–C, the patients have residual tumors at the time of lymphocyte infusion in a clinical setting, and it is conceivable that the donor-derived immune system may acquire tolerance to TAAs. To examine whether the tumor-bearing condition in donors influences the antitumor effect of HSCT, transplantation was performed with BMs and lymphocytes derived from the tumor-bearing donor mice. A tetramer assay showed that the number of AH-1^+^ CD8^+^ T cells was significantly increased in tumor-bearing mice compared with naïve mice (Fig. 2A), indicating that the presence of CT26 tumor cells primes and increases the tumor-specific lymphocytes.

We then examined whether the increase of tumor-specific CD8^+^ T cells in the donors results in the antitumor effects in the recipient mice. The growth of tumors was substantially suppressed in the HSCT mice infused with the lymphocytes of tumor-bearing donors compared with that in HSCT mice with a naïve donor (Fig. 2B), suggesting that a fraction of donor lymphocytes from tumor-bearing mice, which are primed in response to TAAs, remains responsive upon transplantation. The survival of HSCT mice infused with lymphocytes of tumor-bearing donors was significantly prolonged compared with that with lymphocytes of naïve donors (Fig. 2C). The results suggested that the tumor-bearing status of donors can enhance an antitumor immunity in the HSCT recipient mice.

### Intratumoral IFN-α gene transfer increased tumor-responsive immune cells

**Figure 3:**
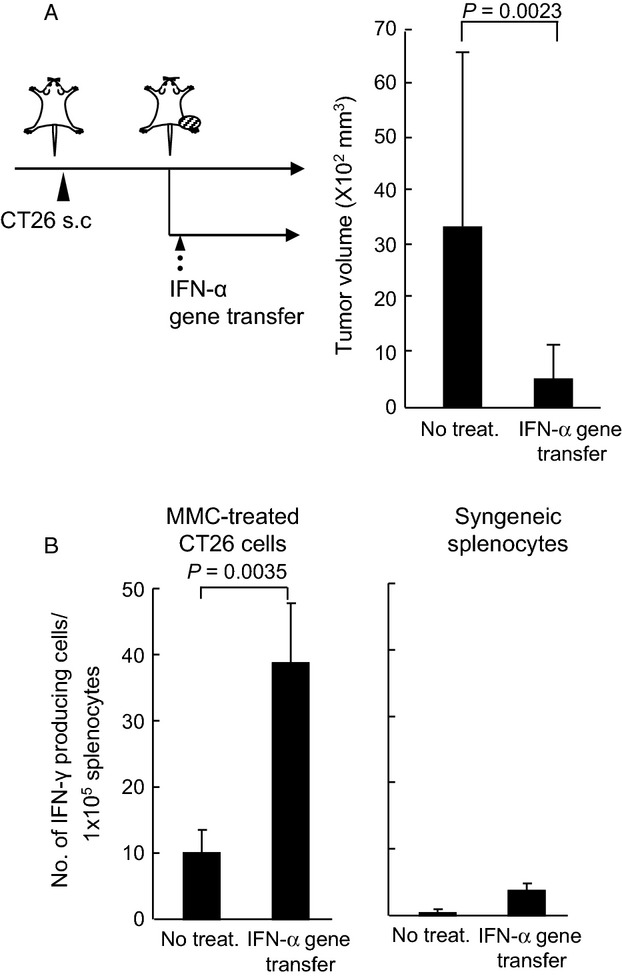
Intratumoral IFN-α gene transfer increased CT26-responsive lymphocytes. (A) Growth suppression of subcutaneous tumors by injection of IFN-α-expressing adenovirus. When CT26 subcutaneous tumors were established, 5 × 10^7^ PFU of Ad-mIFN or control vector (Ad-AP) were injected once into the tumors (*n* = 4). Tumor size was measured 2 weeks after the virus injection. (B) The increase of IFN-γ-positive cells in response to stimulation of CT26 cells by intratumoral IFN-α gene transfer. Two weeks after virus injection, splenocytes were isolated from mice, and cocultured with CT26 cells or control lymphocytes (*n* = 3). IFN-γ-positive cells were analyzed by ELISpot assay. IFN, interferon; Ad-mIFN, adenovirus vectors expressing mouse interferon-α; Ad-AP, adenovirus vectors expressing alkaline phosphatase.

The antitumor effect induced by HSCT appears to be affected by the priming status of donor lymphocytes to TAAs, as shown in Figure 2. We then examined whether a further increase of primed T cells in the donors enhances the antitumor immunity in HSCT recipients. We previously reported that the expression of type I IFN in the tumors augments the maturation of antigen presenting cells and enhances their antigen presentation capacity, and effectively induces systemic antitumor immunity in several animal models^11^,^15^,^19^. In this study also, adenovirus-mediated IFN-α gene transfer significantly suppressed the growth of CT26 subcutaneous tumors (Fig. 3A), and increased the number of tumor-responsive splenocytes by an ELISpot assay (Fig. 3B), indicating that an intratumoral IFN-α gene transfer further promotes the priming of lymphocytes to TAAs in donor mice.

### Donor preimmunization by intratumoral IFN gene transfer strengthened antitumor immunity of HSCT

**Figure 4:**
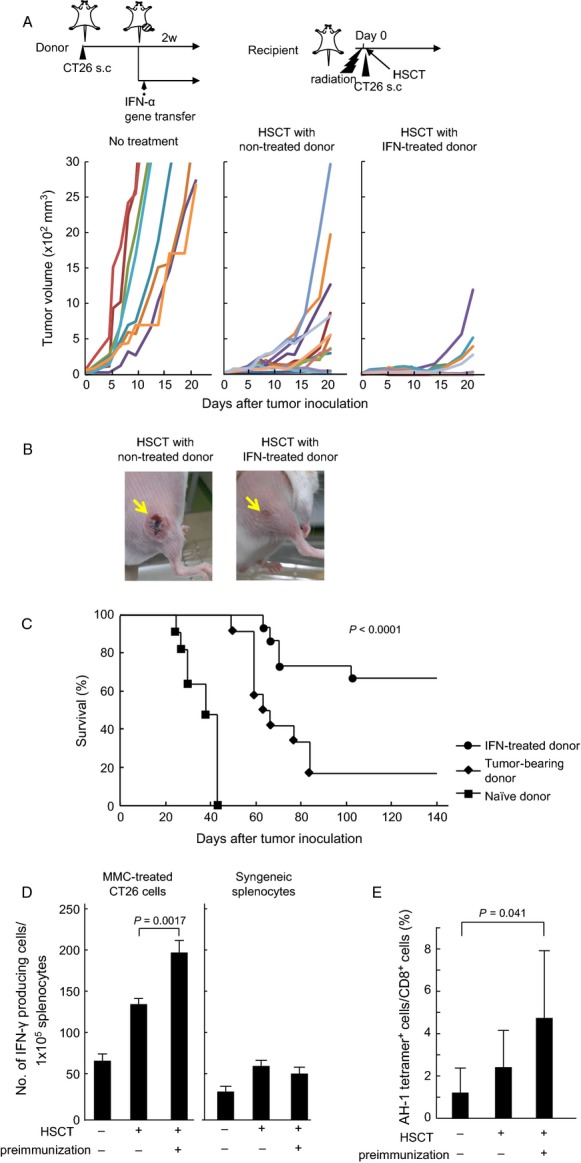
Preimmunization of donors strengthened the antitumor effect of syngeneic HSCT. (A) Growth suppression of subcutaneous tumors in syngeneic HSCT mice with preimmunized lymphocytes. The irradiated BALB/c mice received a infusion of BM and splenic T cells derived from nontreated or IFN-treated tumor-bearing mice. CT26 cells were inoculated into right legs. (B) Photographs of representative tumors 27 days after tumor inoculation. Left: a HSCT mouse infused with lymphocytes isolated from nontreated donors. Right: a HSCT mouse infused with lymphocytes isolated from IFN-treated donors. (C) The preimmunization of donors by IFN gene transfer extended the survival of HSCT mice. Non-HSCT mice: *n* = 5. In HSCT mice, donor lymphocytes were isolated from nontreated tumor-bearing donors (*n* = 12) or IFN-treated tumor-bearing donors (*n* = 14). (D) The increase of IFN-γ-positive cells in HSCT mice with IFN-treated donors. Two weeks after HSCT, splenocytes were isolated from HSCT mice, and cocultured with CT26 cells or control lymphocytes (*n* = 3). IFN-γ-positive cells were analyzed by ELISpot assay. (E) The increase of tumor-specific CD8^+^ T cells in HSCT mice with IFN-treated donors. The splenocytes were isolated 2 weeks after HSCT, and CT26-specific AH-1-tetramer-positive cells were analyzed by flow cytometry (*n* = 4). HSCT, hematopoietic stem cell transplantation; BM, bone marrow; IFN, interferon.

We next examined whether the preimmunization of donor lymphocytes by intratumoral IFN gene transfer more enhances the antitumor effect of syngeneic HSCT. The HSCT with lymphocytes, which were isolated from nontreated tumor-bearing donors, significantly suppressed the CT26 tumor growth of recipient mice compared with that in control mice and tumors disappeared in two of 12 mice (16.7%), whereas HSCT with lymphocytes, which were isolated from donors treated with intratumoral IFN gene transfer, markedly suppressed the tumor growth and tumors disappeared in nine of 14 mice (64.3%) (Fig. 4A). In all of the cured mice, the tumors that were once formed until 7 days after HSCT, became gradually smaller, then changed to an ulcer-like lesion (Fig. 4B) and finally disappeared. The survival of HSCT mice transferred with IFN-treated donor lymphocytes was also significantly prolonged compared with that of HSCT mice infused with nontreated lymphocytes (Fig. 4C).

An ELISpot assay showed that the number of IFN-γ^+^ spots in response to CT26 cells was significantly increased in HSCT mice infused with IFN-treated donor lymphocytes as compared to HSCT mice infused with nontreated lymphocytes (Fig. 4D). A tetramer assay also showed that HSCT with IFN-treated lymphocytes increased the number of AH-1^+^ CD8^+^ T cells more than HSCT with nontreated lymphocytes did (Fig. 4E). The results suggested that the promotion of priming to TAAs of donor lymphocytes by intratumoral IFN-α gene transfer markedly strengthens the induction of antitumor immunity by syngeneic HSCT.

## Discussion

In autologous HSCT recipients, lymphopenia-induced HP of T cells skews the T-cell repertoire by engaging TAAs, and leads to an induction of antitumor immunity. Baccala et al.^6^ hypothesized that an immunologically delicate environment decreases the activation threshold of tumor-specific T cells by TAA-presenting DCs in the draining lymph nodes, leading to their preferential engagement and expansion. Therefore, lymphopenic conditions are able to create an environment to enhance an antitumor immunity of various immunotherapies through an expansion of tumor-responsive T cells. In fact, there have been several animal studies showing the potential efficacy of gene- and cell-based immunotherapy in syngeneic HSCT mice. The vaccination with syngeneic tumor cells expressing granulocyte macrophage colony-stimulating factor (GM-CSF) showed a strong antitumor effect in the transplanted mice^2^. An immunization with DCs pulsed with whole tumor cell lysates led to efficient antitumor responses in a mouse breast tumor model^20^. Adoptive transfer of tumor-specific T cells has also enhanced antitumor immune responses in lymphopenic mice^5^. We also reported that the combination of immune gene therapies such as allogeneic MHC gene transfer and type I IFN gene transfer in the early period after syngeneic HSCT induces a systemic antitumor immunity^12^,^13^,^21^. However, the development of other immunotherapeutic strategies is necessary to successfully eradicate preexisting malignant tumors. In this study, we demonstrated that the preimmunization of donor lymphocytes clearly enhances the efficacy of syngeneic HSCT.

Although it was conceivable that lymphocytes acquired tolerance to TAAs in tumor-bearing donors and that a reconstituted immune system in the recipient mice may not be able to induce an immune response to TAAs, HSCT with the lymphocytes of tumor-bearing donors more strongly suppressed the growth of CT26 tumors compared with HSCT with naïve donors did, demonstrating that a tumor-bearing condition in the donors is not the limitation of HP-mediated tumor immunity. Lymphodepletion can eradicate suppressive immune cells in the host, including Tregs and myeloid-derived suppressor cells^22^–^24^, and the repopulation of tumor-specific effector T cells significantly exceeded that of Tregs, as the expansion of Tregs was limited by interleukin (IL)-2 availability^25^. Brody et al.^7^ also reported that the transplantation increases the ratio of effector T cells/regulatory T cells in the transferred splenocytes, due to the synergistic effect of the HP combined with encountering their cognate antigen in tumor-bearing mice. In addition, we recently found that HSCT significantly decreased the frequency of Tregs per CD4^+^ T cells in the tumors but not in the spleens, and that the blockade of Tregs was dependent on IL-6 produced by DCs in the tumors^8^. The break of regional immune tolerance within tumors may be another mechanism of an antitumor immunity of HSCT. The integrated mechanisms may contribute to a strong antitumor effect by HSCT with primed donor lymphocytes without the need for ex vivo expansion of tumor-specific T cells.

In this study, we employed an intratumoral IFN gene transfer as the preimmunization method. Regarding other preimmunization strategies, Filatenkov et al.^26^ vaccinated the donors with a subcutaneous injection of irradiated tumor cells and CpG-enriched oligonucleotides, which are adjuvants that stimulate DCs via TLR-9, and Brody et al.^7^ described in situ vaccination combining chemotherapy with intratumoral injection of CpG in murine models. Borrello et al. reported a phase 2 trial of GM-CSF-secreting cellular immunotherapy in combination with autologous HSCT as postremission therapy for acute myeloid leukemia. In this trial, primed lymphocytes were collected after pretransplantation immunotherapy and reinfused with the stem cell graft, resulting in the improvement of overall survival^14^. As tumor-reactive T cells are mostly polyclonal, and heterogeneous expressions of various TAAs coexist even in a tumor mass, the in vivo stimulation of multiple tumor-reactive lymphocytes by these preimmunization strategies may overcome the problem of clonal limitation in the adaptive transfer of a particular TAA-specific T cell. Compared with the previous approaches, a major advantage of an in vivo type I IFN gene transfer is that it has local effects on tumor sites: IFN significantly induces cell death and growth inhibition^11^,^15^,^19^. In addition, IFN gene transfer has direct effects upon DCs such as the maturation of the cells and the production of immune-stimulatory cytokines and the enhancement of inhibitory activity against Tregs^12^. Therefore, preimmunization by a local IFN gene therapy is a promising therapeutic strategy, especially in a case where cancer conditions need strong local tumor control and systemic antitumor activity.

Allogeneic HSCT has been applied not only for hematologic malignancies but also for solid cancers, such as renal and breast cancers^1^,^27^–^29^. However, an important limitation of allogeneic HSCT is the development of graft-versus-host disease (GVHD), which occurs in a severe form in 30–50% of patients who received this therapy^30^. On the other hand, the procedure of autologous HSCT is well established and can be done safely with a mortality rate of <5%, which is much lower than the allogeneic HSCT. In addition, although the donor immunization in allogeneic HSCT may have an ethical issue in a clinical setting, it is a highly feasible approach in autologous HSCT. As, at present, autologous HSCT is clinically practiced after intensive chemotherapy in patients with lymphomas and solid cancers such as neuroblastoma and sarcoma^1^, the infusion of hematopoietic stem cells and preimmunized lymphocytes after high dose chemotherapy is an attractive clinical introduction of HSCT-mediated immunotherapy in such patients.

In conclusion, the antitumor immunity induced by HSCT is dependent on the priming status of donor lymphocytes to TAAs, and the combination of HSCT with preimmunization by intratumoral type I IFN gene transfer can induce a strong antitumor immunity. This therapeutic strategy deserves an evaluation in future clinical trial for solid cancers.
